# Why the Ala-His-His Peptide Is an Appropriate Scaffold to Remove and Redox Silence Copper Ions from the Alzheimer’s-Related Aβ Peptide

**DOI:** 10.3390/biom12101327

**Published:** 2022-09-20

**Authors:** Paulina Gonzalez, Laurent Sabater, Emilie Mathieu, Peter Faller, Christelle Hureau

**Affiliations:** 1 LCC-CNRS, Université de Toulouse, CNRS, 31077 Toulouse, France; 2 Laboratory of Biometals and Biological Chemistry, Institut de Chimie (UMR 7177), Université de Strasbourg-CNRS, 4 rue Blaise Pascal, 67000 Strasbourg, France

**Keywords:** ATCUN, copper, amyloid-β, peptides, ROS, kinetics, drug candidates

## Abstract

The progressive, neurodegenerative Alzheimer’s disease (AD) is the most widespread dementia. Due to the ageing of the population and the current lack of molecules able to prevent or stop the disease, AD will be even more impactful for society in the future. AD is a multifactorial disease, and, among other factors, metal ions have been regarded as potential therapeutic targets. This is the case for the redox-competent Cu ions involved in the production of reactive oxygen species (ROS) when bound to the Alzheimer-related Aβ peptide, a process that contributes to the overall oxidative stress and inflammation observed in AD. Here, we made use of peptide ligands to stop the Cu(Aβ)-induced ROS production and we showed why the AHH sequence is fully appropriate, while the two parents, AH and AAH, are not. The AHH peptide keeps its beneficial ability against Cu(Aβ)-induced ROS, even in the presence of Zn^II^-competing ions and other biologically relevant ions. The detailed kinetic mechanism by which AHH could exert its action against Cu(Aβ)-induced ROS is also proposed.

## 1. Introduction

Alzheimer’s disease (AD) is a progressive, neurodegenerative disorder affecting neurons in specific places in the brain, causing cognitive impairment and disability in daily life activities. It represents more than half of all dementia cases, which are listed as the world’s seventh leading cause of death [1,2,3,4,5]. The number of patients suffering from dementia is 55 million, a number that will dramatically increase in the near future (a double-to-triple increase is expected on the horizon of 2050). This is due, first, to the ageing of the population, but also to the lack of disease-modifying drugs, id est, molecules able not only to relieve symptoms, but also to intervene in the neurodegenerative process. 

AD is multifactorial and several therapeutic targets are currently being investigated [6,7,8,9]. This is the case for instance of the amyloid-β (Aβ) peptide [5,10] and the hyperphosphorylated Tau protein [11,12] that can assemble to form extracellular amyloid deposits and intracellular tangles, respectively. In the present article, we focus on the redox-competent copper (Cu) ions [13,14,15,16,17,18]. Cu ions have been shown to modify the assembly properties of Aβ [19]. Beyond that, Cu ions have the ability to generate Reactive Oxygen Species (ROS) when bound to Aβ that contribute to the overall oxidative stress observed in AD in relation with neuro-inflammation [20,21]. Cu(Aβ)-catalyzed incomplete reduction of dioxygen fueled by biological reductants such as ascorbate (Asc) leading to superoxide, [22] hydrogen peroxide [23] and hydroxyl radicals [24,25] have been reported. As a direct consequence, we and others have developed therapeutic approaches targeting Cu ions with the aim to extract them from Aβ and to redox silence them [13,14,15,16,17].

Here, we make use of a short peptide, namely the Ala-His-His-COOH (AHH), to illustrate why such peptide scaffolds are highly suitable to stop the production of ROS induced by Cu(Aβ). The main drawback of peptides, id est, a weak proteolytic stability [26], is currently the subject of intense lines of research aimed at overcoming such issues, either by the use of peptidomimetic molecules [27,28,29], D-isomers, [30] or smart delivery systems [31,32,33,34]. In contrast, peptides have the great advantage of being easily tunable. For instance, cell-penetrating peptides (CPP) [35], including brain-penetrating sequences [36], could easily be appended to improve blood–brain barrier entrance. Another possibility would be the combination with peptide-based scaffolds built on poly(His) [37] and poly(Glu) [38] and recently reported as drug delivery vehicles. Hence, peptides are fully appropriate drug candidates. 

A key point to consider is the Cu versus Zn selectivity of the intended peptide sequences. Indeed Zn^II^ is present in the synaptic cleft at concentrations much higher (about 10 to 100-fold) than Cu and can thus interfere with Cu detoxification [39]. Both ions have been reported to bind to the N-terminal part of the Aβ peptide (having the sequence DAEFRGHDSGYEVHHQK, id est, H_2_N-Asp-Ala-Glu-Phe-Arg-His-Asp-Ser-Gly-Tyr-Glu-Cal-His-His-Gln-Lys-COOH, see Scheme S1, named Aβ_1–16_, where the full-length peptides are 40- and 42-amino-acids-long: Aβ_1–40/42_, here, Aβ_1–16_, is noted Aβ, but with distinct binding sites [39,40,41]. At neutral pH, Cu^II^ is bound to the terminal amine, two imidazole rings from two of the three His at positions 6, 13, and 14, and the peptide carbonyl function of Asp1-Ala2, [42] while Zn^II^ is bound by two imidazole rings from the His and two carboxylate (from Asp1, Glu3, Asp7, or Glu11) [43] and Cu^I^ by two imidazole rings from the His [44,45]. The Aβ affinity of Cu^II^ is about 10^9^ M^−1^ [46] that of Zn^II^ 10^5^ M^−1^ [47], while for Cu^I^, this is still under debate, with values ranging from 10^6^ to 10^10^ M^−1^ [48,49,50]. Hence, the Cu over Zn selectivity of the intended peptide ligand should overcome that of Aβ, that is, 10^4^ in case of Cu^II^. 

**Scheme 1:**
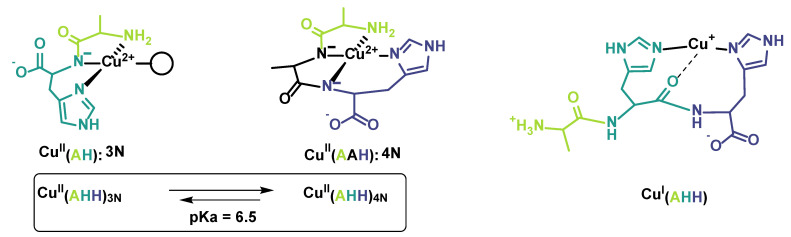
Cu^II^-binding sites in AH, AAH at neutral pH, and pH-dependent equilibrium of Cu(AHH). Cu^I^-binding site in AHH (proposed based on literature data, the participation of the -CO from the peptide bond is still under debate) [48,49,50,74,75].

Cu^II^ binding to the short AHH peptide was previously studied using a set of spectroscopy and it was shown that it has the ability to bind Cu^II^ in two different sites at neutral pH (Scheme 1) [51]. The AHH peptide is issued from AH and AAH that were also studied here for comparison purposes. AH is a canonical Cu^II^-binding motif found in the wound-healing factor GHK peptide involved in anti-inflammation process [52,53,54] and binds to Cu^II^ via the terminal amine, the His imidazole ring, and the deprotonated amide in between, thus leaving a labile site on the fourth equatorial position (Scheme 1, 3N coordination mode) [55]. AAH contains the ATCUN (Amino-Terminal CU and Ni-binding) motif, which, by definition, has one histidine in the third position and a free terminal amine [56,57,58,59]. In ATCUN peptides, Cu^II^ is bound by the terminal amine, the His imidazole ring. and the two deprotonated amides in between (Scheme 1, 4N coordination mode) [55]. In AHH, having His at both positions 2 and 3, the switch between the 3N and 4N motifs can be driven by pH [51] and by the addition of external ligands such as imidazole, in the presence of which the Cu^II^ complexes equilibrated between 4N and a 3N + 1N coordination mode [60]. The three peptides may fulfill the selectivity criteria, since Cu^II^ affinity lies in the 10^12^–10^14^ M^−1^ range for such motifs [56,57,58,59,61,62], while Zn^II^ affinity is expected to be weaker than the one of Aβ. Indeed, there are fewer than three Zn^II^ binding atoms in the peptides, since Zn^II^ is not able to induce the deprotonation of the peptide bond in contrast to Cu^II^, which is the event leading to the huge affinity observed in the case of Cu^II^ [63,64].

In the present article, we report on the ability of the chimera peptide AHH to stop Cu(Aβ)-produced ROS using the ascorbate consumption assay as a leading method, and we tentatively propose a mechanism of action. ATCUN peptides and their derivatives have been used in several biomedical applications [65,66], including against AD [67,68,69,70,71,72,73]. The added value of the AHH peptide is discussed later in the text. 

## 2. Results and Discussion

### 2.1. Effect on Cu- and Cu(Aβ)-Induced ROS Production

**Figure 1:**
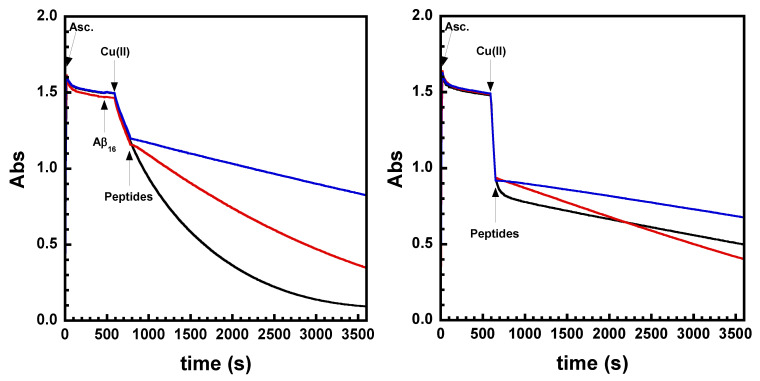
Ascorbate consumption (measured at λ = 265 nm) as a function of time. Ascorbate was added first, followed by Aβ_1–16_ (right panel) after 6 min, and Cu^II^ after 10 min, and the peptides (AH in red, AAH in black, and AHH in blue) when an absorbance of 1.0 (**left**) or 1.2 (**right**) was reached. Measurements were performed in 50 mM HEPES buffer (pH 7.5) at 25 °C with continuous stirring. Final concentrations: [Asc] = 100 µM, [Aβ] = 12 µM, [Peptide] = 12 µM, Cu^II^ = 10 µM.

The effect of AH, AAH, and AHH on the Cu- and Cu(Aβ)-induced ROS production was investigated by following ascorbate (Asc) consumption by UV–Visible spectroscopy at 265 nm (ε_Asc_ = 14,500 M^−1^.cm^−1^) (Figure 1 and Figure S1). It was previously shown that, because Asc fuels the production of ROS, following its consumption is a straightforward way to monitor the formation of ROS production, H_2_O_2_, or HO° [76,77]. Hence, this is a standard and well-accepted method [16], with less bias than other ROS detection assays [78]. Two kinds of experiments were performed: (i) Asc is added once the peptide (AH, AAH, and AHH, noted pep unless specified) has been mixed with Cu^II^ or Cu^II^(Aβ) (Figure S1) or (ii) the peptide is added during the course of Asc consumption triggered by Cu^II^ or Cu^II^(Aβ) (Figure 1). With the first experiment (Figure S1), Cu^II^ or Cu^II^(Aβ) and the peptides are premixed in solution before the addition of Asc to evidence differences in the redox properties of the Cu^II^(pep) complexes (when added to Cu^II^) and the peptide ability to remove Cu^II^ from Aβ (when added to Cu^II^(Aβ)). With the second experiment (Figure 1), the kinetics of Cu^II^ complexation are additionally probed; indeed, there is a competition between the reduction of Cu^II^ or Cu^II^(Aβ) by Asc and the Cu^II^ uptake by the ligand [79,80].

With Cu (in absence of Aβ), AH displays a steeper slope than AAH and AHH, indicating that AH is not able to stop Cu-induced Asc consumption (Figure 1 and Figure S1, red lines, left). This is in accordance with the ability of AH to bind Cu^II^ only in a 3N-motif, the resulting complex Cu(AH) being redox-active [55]. AAH is able to prevent or stop Asc consumption in conditions corresponding to pre-mixture or added during Asc consumption, respectively (Figure 1 and Figure S1, black lines, left). This is in line with the formation of a redox-inert Cu(AAH) complex where Cu^II^ is bound in a 4N-motif, which is resistant to reduction by Asc, in line with previously reported electrochemical data [51]. As shown in Figure 1, panel A (between t = 600 and 700 s, black curve), the change in the slope to reach a straight line is not immediate (as for AH and AHH) and a delay is thus observed between the addition of AAH and the total slowdown of Cu-induced Asc consumption. This indicates slow kinetics of complex formation. AHH displays the best ability to limit Asc consumption in both experiments (Figure 1 and Figure S1, blue lines, left), which suggests a fast complexation of Cu^II^ by the peptide and the subsequent formation of a complex which cannot be reduced by Asc. 

The effect of AH, AAH, and AHH was then investigated on Cu(Aβ)-induced ROS production (Figure 1 and Figure S1, right). Similar to the precedent experiments, the ability of AH, AAH, and AHH to limit Asc consumption was tested in pre-mixed (Figure S1, right) or addition during Asc consumption (Figure 1, right) conditions. The results show the ability of AH to remove Cu from Aβ and slow down Asc consumption, but not completely, due to the ability of Cu^II^(AH) to react with Asc, as shown in the experiments without Aβ being present. AAH was able to stop Asc consumption when AAH was first premixed with Cu(Aβ) (Figure S1, right), but not when it was added during Cu(Aβ)-induced Asc consumption (Figure 1, black lines, right). Since binding of Cu^II^ by AAH is thermodynamically favored compared to Aβ, these results indicate that the slow kinetics of Cu^II^ extraction from Aβ hampered AAH from efficiently halting related Asc consumption. Finally, AHH was able to lessen Asc consumption efficiently regardless of the conditions (Figure 1 and Figure S1, blue lines, right)). The arrest of ROS production is, however, not total. 

**Figure 2:**
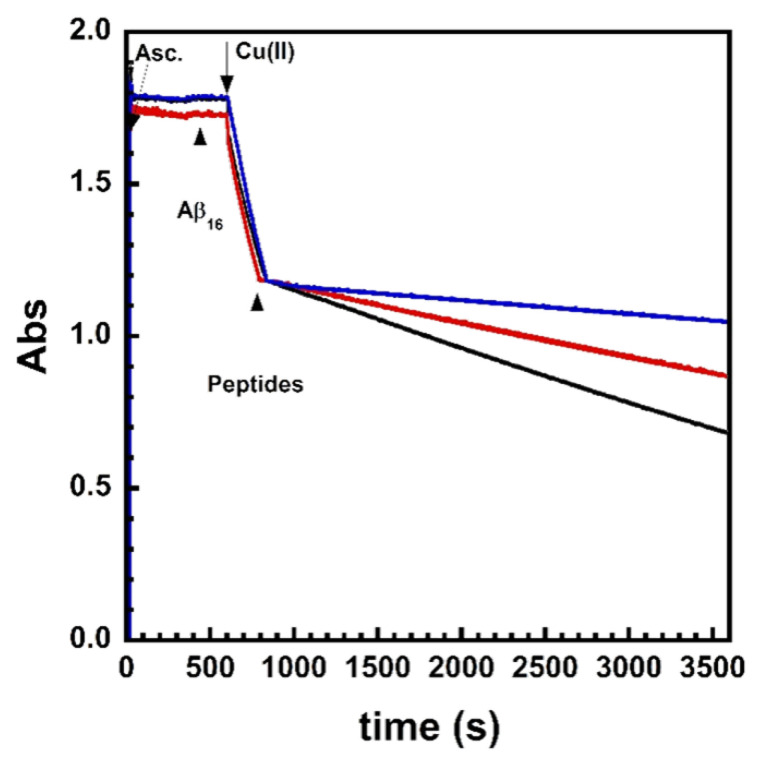
Ascorbate consumption (measured at λ = 265 nm) as a function of time. Ascorbate was added first, followed by by Aβ_1–16_ after 6 min and Cu^II^ after 10 min, and, finally, the AHH peptides at three pH values: 6.8 (black), 7.5 (red), and 8.2 (blue), when an absorbance of 1.2 was reached. Measurements were performed in 50 mM HEPES buffer (pH 7.5) at 25 °C with continuous stirring. Final concentrations: [Asc] = 100 µM, [AHH] = 12 µM, Cu^II^ = 10 µM.

To gain further insights into the mechanism by which AHH lessens Cu^II^/Cu^II^(Aβ)-induced Asc consumption, the experiments were performed at different pH, with (Figure 2) and without Aβ (Figure S2). The effect of AHH on Cu and Cu(Aβ)-induced Asc consumption is pH-dependent: the higher the pH is, the better the arrest of ROS production. The pH-dependent ability of AHH to limit Asc consumption reflects the pH-driven switch of AHH between the 3N and 4N-motifs, since this equilibrium has a pKa of ~6.5. Hence, at pH 6.8, there is about 30% of the complex Cu(AHH) in a redox-competent 3N-motif, while at pH 8.2, >95% of the formed complex is in the 4N form resistant to Asc reduction.

At this stage, we can hypothesize that AHH first binds Cu^II^ in a 3N motif, which would induce kinetics similar to that of AH, and then rearranges in a 4N-redox-inactive motif as AAH. According to this hypothesis, the peptide would combine the two positive effects of the 3N and 4N motifs, which are a fast Cu^II^-binding kinetic, and the formation of a redox-inactive Cu^II^ complex, respectively. To question this hypothesis, we investigated the kinetics of Cu^II^ captured by the peptides by stopped-flow experiments.

### 2.2. Kinetics of Cu and Cu(Aβ) Capture by the Peptides

**Figure 3:**
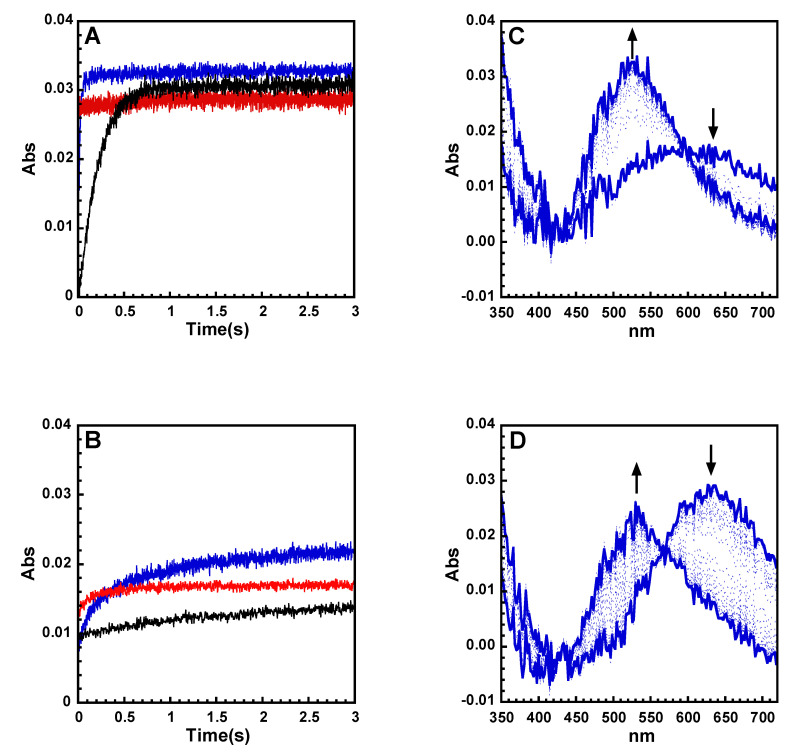
Stopped-flow kinetic traces of Cu^II^ coordination by the peptides AH (red), AAH (black), and AHH (blue) (panel (**A**) (Cu^II^ only); panel (**B**) (Cu^II^(Aβ)) and selection of corresponding UV–Vis spectra (panels (**C**) and (**D**), respectively). The absorption was measured at 590 nm for AH and at 530 nm for AAH and AHH. Measurements were performed in 100 mM HEPES buffer (pH 7.4) at 25 °C. Final concentrations: [Cu^II^] = 450 µM, [peptide] = [Aβ] = 500 µM.

First, stop-flow experiments were performed by mixing Cu^II^ with the peptides and recording absorption spectra with an integration time of 1 ms (Figure 3, Figures S3 and S4). The appearance of the Cu(pep) UV–Vis signature was monitored at 530 nm and 590 nm, corresponding, respectively, to the maximum absorption for Cu(AAH) (4N) and Cu(AH) (3N) species [51]. By the naked eye, it can be seen that the rate of Cu(pep) formation is Cu(AH) > Cu(AHH) > Cu(AAH), in line with the previously described ROS results. Cu(AH) formation is too fast to be measured with this technique and is completed in less than 5 ms, which corresponds to the dead time of the UV–Vis stopped-flow spectrometer. Since several steps are at play (see below), the overall kinetic rates of Cu(AAH) and Cu(AHH) formation were evaluated by the t_1/2_, at which half of the final Cu^II^(pep) complexes are formed at various wavelengths (Table 1). Cu(AHH) formation is about eight times faster (t_1/2_ = 18 ms) than Cu(AAH) formation (t_1/2_ = 150 ms). 

**Scheme 2:**
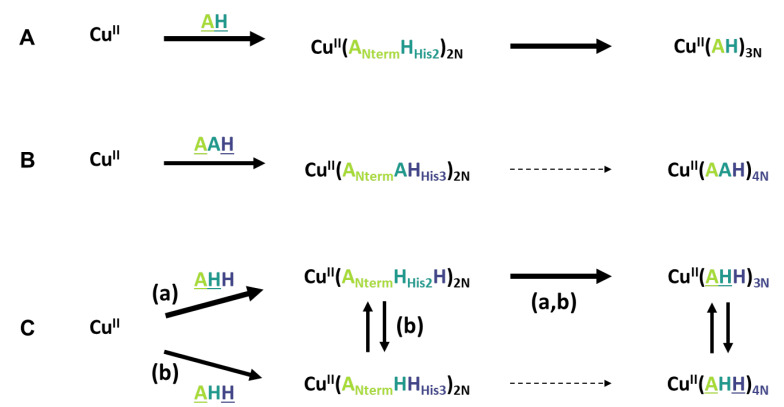
Summary of kinetic data for the Cu^II^ binding by AH (**A**), AAH (**B**), and AHH (**C**). The thickness of the arrows mirrors the rate of the reaction (thicker the arrow, faster the reaction is and dotted arrows correspond to the slowest rate). The His (H) residues underlined in (**C**) correspond to those involved in Cu^II^ anchoring.

The formation of Cu(AAH) is at least 20 times slower than that of Cu(AH). To account for this difference, we have to take into consideration very recent reports on the kinetics of formation of ATCUN Cu^II^ complexes [81,82,83,84,85,86]. It proceeds via several successive steps: anchoring of the Cu^II^ by the imidazole ring of His, then formation of a metallacycle with the anchoring of the N-terminal amine, leading to the 2N intermediate (Scheme S2 for a drawing of the various intermediates described in this work) and rearrangement of the 4N ATCUN motif [81]. The rate-limiting step is the 2N → 4N corresponding to the deprotonation of the peptide bond in between the two anchors. We can thus hypothesize a similar binding pathway for the formation of the Cu(AH) complex, id est, anchoring of the Cu^II^ by the imidazole ring of His, then formation of a metallacycle with the anchoring of the N-terminal amine, leading to the 2N intermediate and formation of the 3N motif. In the case of AH, the various rates would be strongly higher than that of AAH because (i) the smaller size of the metallacycle formed is much more favorable to anchor the N-terminal amine and form the 2N intermediates and (ii) there is only one peptide bond to deprotonate (from 2N to 3N, instead of 4N in the case of AAH) (Scheme 2, panels A and B). In the case of AAH, we do observe the very fast formation of the 2N species absorbing near 700 nm (Figure S3, panel C), reminiscent of those thoroughly described in [81], followed by its evolution to the 4N species, which is the rate-limiting step of the whole reaction.

For the hybrid peptide AHH, the rate of complex formation is in between those of AH and AAH (Figure 3, panel A). As can be seen in Figure 3, panel C, a large absorption band is observed at about 600 nm that forms rapidly (within the dead time of the stopped flow) and that can be attributed to a mixture of 2N and 3N intermediates. Then it evolves, during the rate-limiting step, toward the 4N species with a maximum absorption at 530 nm. The formation of the 2N and/or 3N species is so fast that we cannot distinguish between the two possible pathways (paths a and b in Scheme 2, panel C).

The same experiment was performed in the presence of Aβ. Overall, all the previous rates of Cu(pep) formation are slowed down (Figure 3, panel B and Figure S4). This is in line with (i) the presence of the pre-equilibrium of Cu^II^ to Aβ complexation, leading to a strongly reduced concentration of unbound Cu^II^ in solution (dissociative path, Scheme S3) and/or (ii) the more difficult Cu^II^ anchoring when already bound to Aβ (associative path). Based on a recent report [82], we propose that the dissociation path is predominant. Such a contribution of the dissociation path is even more pronounced at the concentration of ROS experiments (50 times lower than in the stopped-flow experiments). For AHH, Cu^II^ extraction from Aβ is observed concomitantly to the formation of the Cu(AHH) complex in its 4N form detected at a λ_max_ of 530 nm (Figure 3, panel D) and the formation of the Cu(AHH)_4N_ is about 15 times slower than in the absence of Aβ. No intermediate species is detected, as indicated by the presence of the isosbestic point at 560 nm. This is in line with a slowdown of their formation rate (due to a decreased concentration of free Cu^II^ in solution in the presence of Aβ), which thus becomes slower than the conversion to the 4N species. In the presence of Aβ, the formation of the intermediate species thus becomes the rate-limiting step. 

### 2.3. Effect of Zn^II^ on Cu(Aβ)-Induced ROS Production

**Figure 4:**
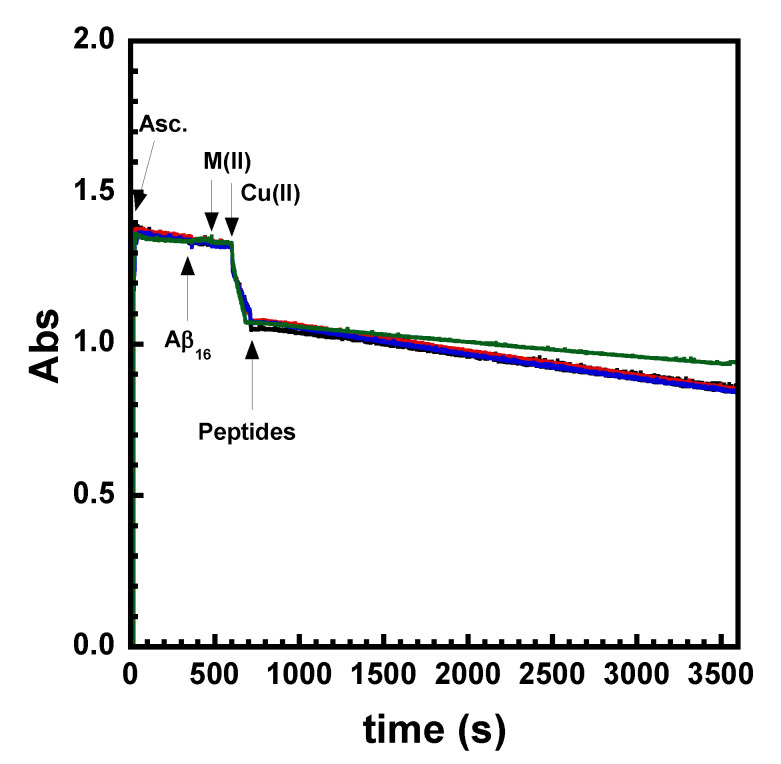
Ascorbate consumption (measured at λ = 265 nm) as a function of time. Ascorbate was added first, followed by Aβ at 6 min, Zn^II^ (green), Mg^II^ (blue), Ca^II^ (red), and no ions (grey) at 10 equiv. versus Cu^II^ at 8 min, and Cu^II^ at 10 min, and, finally, the AHH peptide when an absorbance of 1.2 was reached. Measurements were performed in 50 mM HEPES buffer (pH 7.5) at 25 °C with continuous stirring. Final concentrations: [Asc] = 100 µM, [Aβ] = [peptide] = 12 µM, [Cu^II^] = 10 µM, [Zn^II^] = [Ca^II^] = [Mg^II^] = 100 µM.

In a last part, we investigated how Zn^II^ (and other biologically relevant ions) perturb the arrest of Cu(Aβ)-induced ROS production, as Zn^II^ is one of the metal ions whose content is dysregulated in AD. Asc consumption experiments were performed with an increasing amount of Zn^II^ (0 to 10 eq) (Figure 4, Figures S5 and S6). For the three peptides studied here, an increasing amount of Zn^II^ resulted in a slower Asc consumption. This effect was weak in the case of AHH, since the ROS production rate is already slow, but meaningful, since with Ca^II^ and Mg^II^, no modification is observed (Figure 4). Thus, for the three peptides, Zn^II^ seems to facilitate Cu^II^ binding by the peptides. This is in line with the dissociative pathway we favored previously, where 10 equiv. of Zn^II^ can push Cu^II^ out of the Aβ peptide, but not from the AH, AHH, and AAH ones because the Zn^II^ sites are much weaker than that of the Cu^II^. In other words, based on these results, we proposed a push–pull mechanism in which the Zn^II^ bound to Aβ helps to direct Cu^II^ into the AH, AAH, and AHH ligands.

## 3. Concluding Remarks

**Scheme 3:**
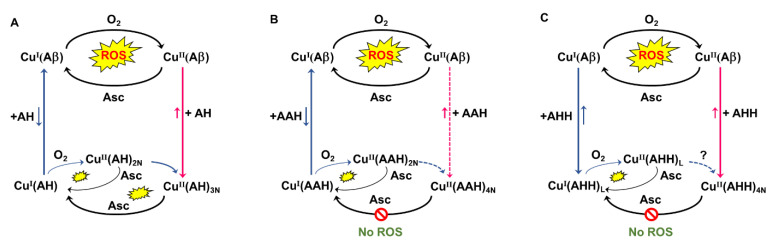
Proposed mechanisms by which the Cu(Aβ)-induced ROS production is impacted by the AH (left, panel (**A**)), AAH (middle, panel (**B**)), and AHH (right, panel (**C**)). Dotted lines indicate slow reactions. The size of the star mirrors the level of ROS formed by the redox couple at play.

A tentative picture of the mode of action of the three peptides under focus can thus be drawn (Scheme 3A–C). Addition of peptides during Cu(Aβ)-induced Asc consumption leads to Cu^II^ extraction from Aβ. This reaction competes with the reduction of Cu^II^(Aβ) to Cu^I^(Aβ) and is fast enough only in the cases of AH and AHH. Once formed, the resulting Cu(AH)_3N_ is weakly competent in ROS production, while Cu(AHH)_4N_ complexes are resistant to Asc reduction. Only the AHH peptide can both remove Cu^II^ from Aβ and form a Cu^II^(AHH) complex resistant to reduction by Asc (and thus is unable to produce ROS). The addition of Zn^II^ improves slightly the effect of the peptides due to an increase in the Cu(Aβ) dissociation pre-equilibrium. This indicates that Zn^II^ selectivity of the AHH peptide is, as explained in the introduction, fully appropriate. 

The presence of two His residues could also give to AHH the additional possibility to compete for Cu^I^ with Aβ, leading an L-motif (Scheme 1), while the Cu^I^ affinity of AH and AAH, containing only one His, is too weak to do so. After oxidation of Cu^I^, the intermediate formed could either rearrange to form the Cu^II^(AHH)_4N_ or be reduced by Asc. To probe the Cu^I^ path (blue lines in Scheme 3), the AAHH peptide was tested (Figure S7). This peptide possesses two His residues and is thus able to bind Cu^I^ in an L-motif, similarly to AHH, and to compete with Aβ for Cu^I^ binding. The results obtained show that AAHH has a weaker ability to arrest Cu(Aβ)-induced ROS formation than AHH. This suggests that the Cu^I^ path might not be the one predominant here. We must be very cautious, since two key reactions occurs in the Cu^I^ path: (1) Cu^I^ extraction from Aβ, which would be similar in case of AHH and AAHH, and (2) the rate of reorganization to the Cu^II^-4N species once the linear Cu^I^ intermediate is oxidized, a parameter that could differ between AHH and AAHH (with a fast enough reorganization in case of AHH but not AAHH). To gain further insights into the mechanism at play during ROS production and to discard the Cu^I^ path, more systematic studies of the impact of sequence modification (number and position of the His residues) on the level of ROS arrest are needed and are currently under investigation in our group. 

In the present study, we have unambiguously shown that the AHH motif is fully appropriate to stop Cu(Aβ)-induced ROS production. AHH combines the two advantageous properties of the two AH and AAH parent ligands, fast Cu^II^ binding, and ability to form a redox-incompetent Cu^II^ complex, respectively. This result underlines the potential of peptide ligands in the search for Cu-targeting therapeutic approaches in the context of AD. It is also interesting to note that for all the previous ATCUN peptides studied, only experiments where the peptide is added to Cu^II^(Aβ) and then the ROS production is triggered by addition of Asc have been reported [67,68,69,70,71,72,73]. Once formed, Cu^II^(ATCUN) complexes have a very low ability to form ROS, as shown here and previously [55,56,87]. Hence, such studies are incomplete. Indeed, in the brain, which is a quite reductive medium [88,89], with up to 300 µM of Asc being present extracellularly, both redox states of the Cu ions are at play and the Cu(Aβ) may be redox cycling. Hence, targeting both Cu^I^ and Cu^II^ ions [90], or having fast enough Cu^II^-complexing agents, are crucial [80,91]. In other words, if ATCUN-based peptides are aimed to be used against Cu(Aβ)-induced ROS formation, the rate of Cu^II^ extraction from Aβ matters and, as we demonstrated, such a kinetic parameter is highly dependent on the exact sequence of the ATCUN peptide chosen. It is here interesting to note that, among the N-terminally truncated forms of Aβ recently put forward [92,93,94,95,96], several of them possess such H_2_N-Xxx-His (3N, Aβ_5–x_) [97,98], H_2_N-Xxx-Zzz-His (4N, Aβ_4–x_, [85,86] Aβ_11–x_ [86]), and H_2_N-Xxx-His-His (hybrid 3N/4N, Aβ_12–x_ [99]) binding sites. Based on our results, the cleavage at position 12 leading to the hybrid 3N/4N binding site may be the most beneficial with respect to Cu(Aβ_1–x_)-induced ROS formation. 

Further works will include modifications of the peptide scaffold to obtain BBB penetration and better metabolic stability. This can be straightforward by appending brain-penetrating sequences and using D-peptide, respectively. To shuttle Cu inside cells is also regarded as an interesting additional property that will overcome the intracellular Cu depletion involved in the disability of secretases to clear Aβ [100,101,102]. This is an exciting follow-up of the present work, which is currently under progress in our team. 

## 4. Experimental Section

### 4.1. Chemicals

Reagents were commercially available and were used as received. HEPES buffer (sodium salt of 2-[4-(2-hydroxyethyl)piperazin-1-yl]ethanesulfonic acid) was bought from Fluka (bioluminescence grade).

The Cu^II^ and Zn^II^ sources were Zn(SO_4_)(H_2_O) and CuCl_2_(H_2_O)_2_ and purchased from Sigma. The concentration of Cu^II^ was determined by dissolving the Cu salt in Mili-Q water to prepare a 100 mM solution considering an extinction coefficient of ε = 12 M^−1^cm^−1^. The concentration of Zn^II^ was determined by weight.

A stock solution (100 mM) of ascorbate was prepared in Milli-Q water at room temperature just before beginning the experiment.

### 4.2. Peptides

Aβ_1–16_ peptide (sequence DAEFRHDSGYEVHHQK, H_2_N-Asp-Ala-Glu-Phe-Arg-His-Asp-Ser-Gly-Tyr-Glu-Cal-His-His-Gln-Lys-COOH, with the three-letter code and referred to as Aβ in the text unless specified, see Scheme 1) was bought from GeneCust (Dudelange, Luxembourg) with a purity grade >98%. Stock solutions of the Aβ peptide were prepared by dissolving the powder in Milli-Q water (resulting pH ~2) at approx. 10 mM. Peptide concentration was then determined by UV–Visible absorption of Tyr10 considered as free tyrosine (at pH 2, (ε_276_–ε_296_) = 1410 M^−1^ cm^−1^). The solutions were diluted down to the appropriate concentration.

### 4.3. Synthesis of Peptides

The AHH, AAH, and AH peptides were synthesized manually with standard 9-fluorenylmethoxycarbonyl (Fmoc) Chemistry on a Fmoc-L-His(Trt)-Wang resin (0.63 mmol/g from Iris Biotech GMBH), through solid-phase peptide synthesis protocols, as previously reported [51].

Stock solutions of peptides were prepared by dissolving the peptides in Mili-Q water. Concentrations were determined by Cu^II^ titration followed by UV–Vis absorption spectroscopy.

### 4.4. UV–Visible Spectroscopy

UV–Vis experiments were performed on a Cary-60 UV–Vis spectrometer at 25 °C with constant stirring.

### 4.5. Ascorbate Consumption Assay

Ascorbate consumption was monitored by UV–Vis. Intensity of the Asc absorption band at λ = 265 nm (ε = 14 500 M^−1^ cm^−1^) was monitored as a function of time.

### 4.6. Stopped-Flow Experiments

Rapid-mixing UV–Vis spectroscopy was carried out using an SFM-20 two-syringe stopped-flow from Biologic combined with a diode array spectrometer composed of a TIDAS J&M MMS-UV/VIS 500-3 detector and a light source HAMAMATSU L7893 light source incorporating a deuterium and a tungsten lamp with optic fibers. Data acquisition, extraction, and treatment were realized with Bio-Kine software. The syringes (Hamilton) were mounted on a rigid drive platform ensuring that the flow was stopped precisely and instantaneously. The contents of the two syringes were rapidly mixed in the mixing chamber and the absorbance of the system recorded over time as full spectra at designated time delays. Typically, one syringe was filled with a solution of peptide at 1mM in HEPES buffer (200 mM, pH 7.4), and the other one was filled with a solution of CuSO_4_ in water at 0.9 mM. An equal quantity of the two solutions were mixed to reach a final concentration of Cu^II^ of 450 µM and peptide of 500 µM in HEPES buffer (100 mM, pH 7.4). The optical length of the UV cuvette is 1 cm.

## Figures and Tables

**Table 1:** Parameters for the kinetics of formation of Cu^II^–peptide complex.

Conditions [a]	*t_1/2_* [ms]	Corresponding Mainly to
AH + Cu	<5	/
AAH + Cu	150 ± 20	2N to 4N conversion
AHH + Cu	18 ± 20	2N and/or 3N to 4N conversion
AHH + Cu(Aβ)	250 ± 50	Formation of 2N and/or 3N intermediates

## Data Availability

Not applicable.

## Supplementary Materials

The following supporting information can be downloaded at: https://www.mdpi.com/article/10.3390/biom12101327/s1, Figure S1: Ascorbate consumption of Cu^II^ and Cu^II^(Aβ) plus the three AH, AAH and AHH peptides; Figure S2: Ascorbate consumption of Cu^II^(AHH) and Cu^II/I^ plus AHH peptide as a function of pH; Figure S3: Stopped-flow kinetic measurements of Cu^II^ coordination by AH, AAH and AHH peptides; Figure S4: Stopped-flow kinetic measurements of Cu^II^(Aβ) capture removal by AH, AAH and AHH peptides; Figure S5: Ascorbate consumption of Cu^II^ plus the three AH, AAH and AHH peptides in presence of increasing amounts of Zn^II^; Figure S6: Ascorbate consumption of Cu^II^ plus the AH and AAH peptides in presence of various dications; Figure S7: Ascorbate consumption of Cu^II^(Aβ) plus the four AH, AAH, AHH and AAHH peptides; Scheme S1: Proposed coordination sites in the intermediates 2N forms for the AH, AAH and AHH peptides; Scheme S2. Proposed mechanism corresponding to Cu^II^ capture out from Aβ by AH (A), AAH (B) and AHH (C); Scheme S3. Proposed coordination sites in the ternary species obtained upon AH and AHH addition to Cu^II^(Aβ) [60,62,82].
