# Supplementary material for: Why the Ala-His-His Peptide Is an Appropriate Scaffold to Remove and Redox Silence Copper Ions from the Alzheimer’s-Related Aβ Peptide
Source: Biomolecules. 2022 Sep 20;12(10):1327. doi: 10.3390/biom12101327 (PMC9599918; doi:10.3390/biom12101327)
Supplement: Supplementary file 1 [file biomolecules-12-01327-s001.zip › biomolecules-1900299-supplementary-edited.pdf]

Why Ala-His-His peptide is an appropriate scaffold to remove and redox silence copper ions from the Alzheimer's related Aβ peptide

SUPPORTING INFORMATION

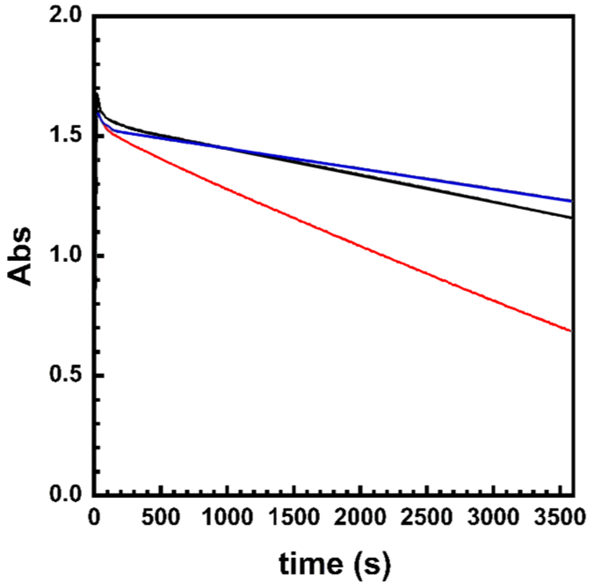

Figure S1. Ascorbate consumption (measured at λ = 265 nm) as a function of time. Aβ (right panel), Cu^{II} and the peptides (AH in red, AAH in black and AHH in blue) were mixed during ten minutes and then ascorbate was added. Measurements were performed in 50 mM HEPES buffer (pH 7.5) at 25 °C with continuous stirring. Final concentrations: [Asc] = 100 µM, [Aβ] = 12 µM, [Peptide] = 12 µM, Cu^{II} = 10 µM.

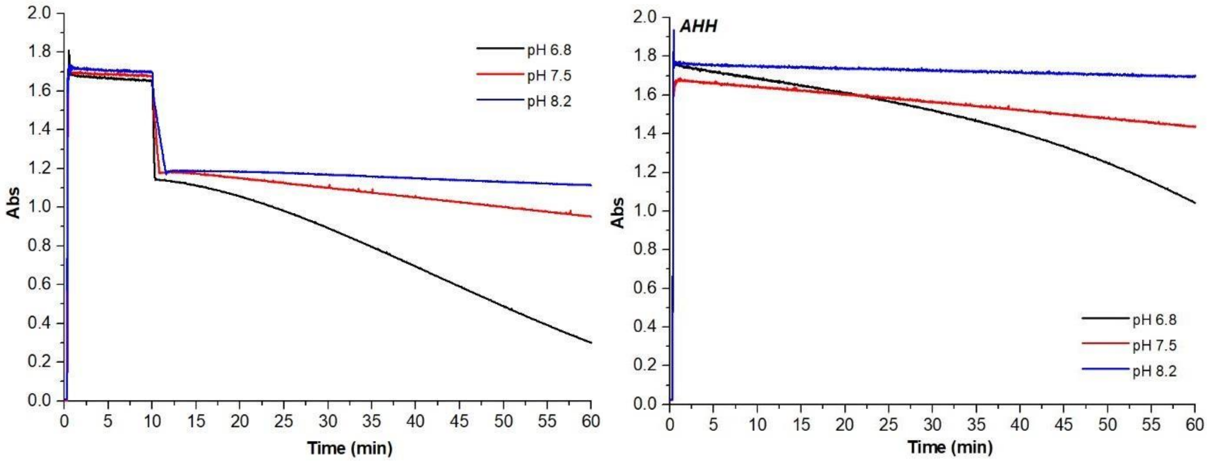

Figure S2. Ascorbate consumption (measured at λ = 265 nm) as a function of time. Left panel: Ascorbate was added first, followed by Cu^{II} after 10 minutes, and the AHH peptides at three pH values: 6.8 (black), 7.5 (red) and 8.2 (blue) when an absorbance of 1.2 was reached. Right panel: Cu^{II} and the AHH peptide were mixed during 10 minutes at three pH values: 6.8 (black), 7.5 (red) and 8.2 (blue) and then ascorbate was added. Measurements were performed in 50 mM HEPES buffer (pH 7.5) at 25 °C with continuous stirring. Final concentrations: [Asc] = 100 µM, [AHH] = 12 µM, Cu^{II} = 10 µM.

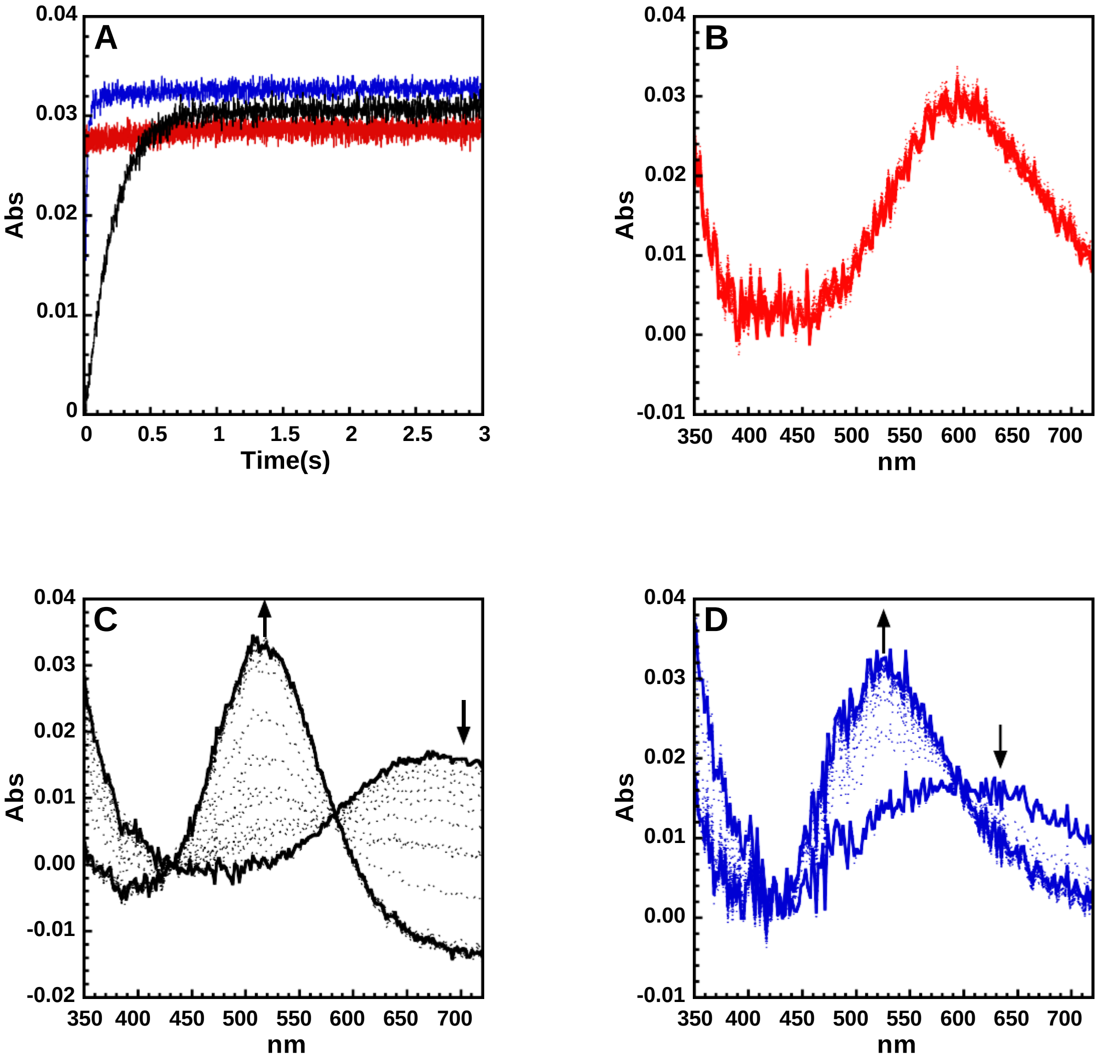

Figure S3. Panel A: Stopped-flow Kinetic traces of Cu^{II} coordination by the peptides AH (red), AAH (black) and AHH (blue) and selection of corresponding UV-Vis spectra in case of AH (red, panel B), AAH (black, panel C) and AHH (blue, panel D). For AH peptide, the absorption was measured at 590 nm, for AAH and AHH at 530 nm. Measurements were performed in 100 HEPES buffer (pH 7.4) at 25 °C. Final concentrations: [Cu^{II}] = 450 µM, [peptide] = 500 µM.

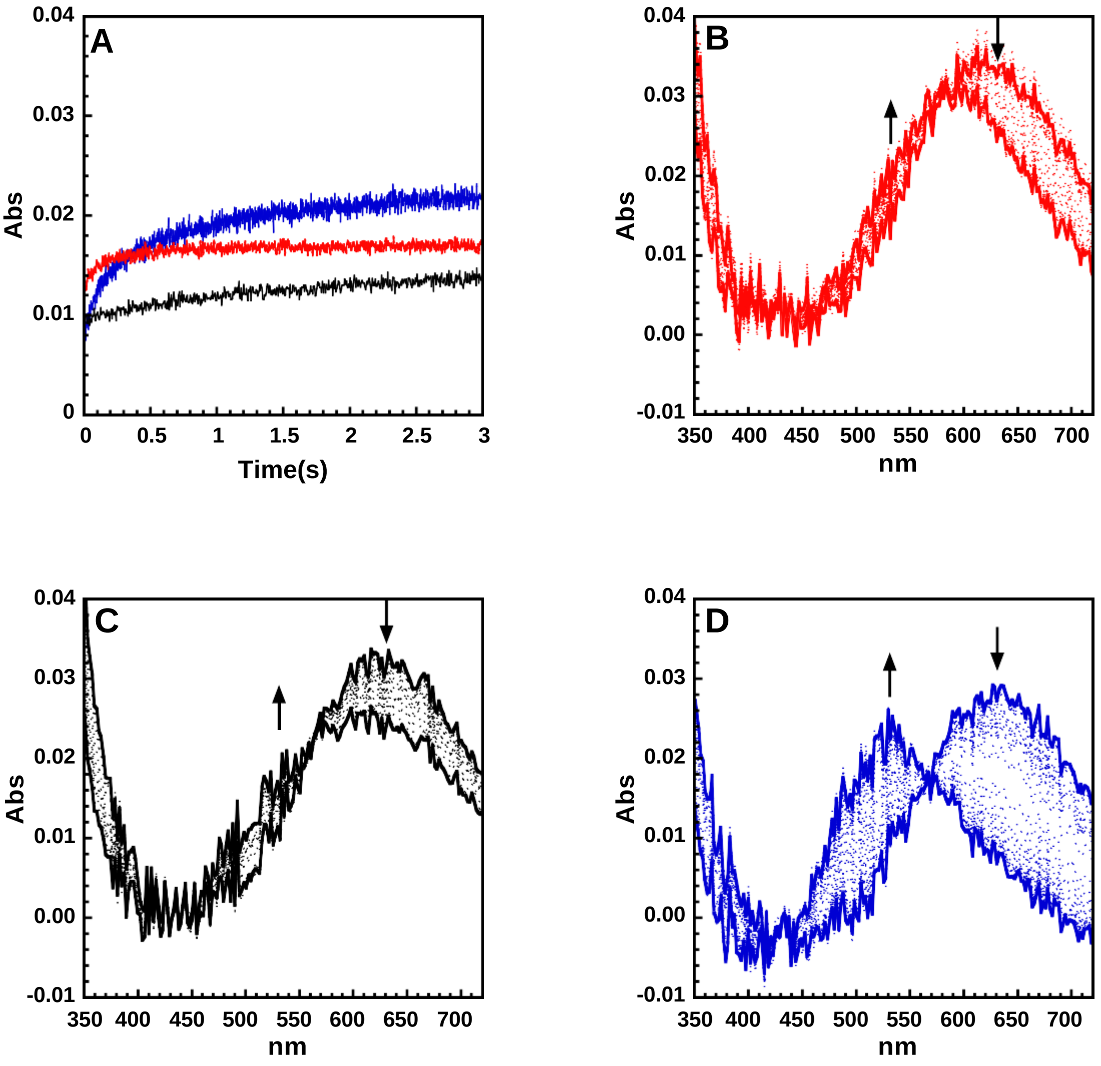

Figure S4. Panel A:Stopped-flow Kinetic traces of Cu^{II} extraction from Cu^{II}(Aβ) by the peptides AH (red), AAH (black) and AHH (blue) and selection of corresponding UV-Vis spectra in case of AH (red, panel B), AAH (black, panel C) and AHH (blue, panel D). For AH, AAH and AHH peptides, the absorption was measured at 590 nm, at 530 nm. Measurements were performed in 100mM HEPES buffer (pH 7.4) at 25 °C. Final concentrations: [Cu^{II}] = 450 µM, [peptide] = [Aβ] = 500 µM.

For AH, the presence of Aβ leads to a decrease in the rate of the overall reaction, as we can observe a small increase during the 0-0.2 s in presence of Aβ corresponding to the end of the $Cu^{II}(AH)_{3N}$ complex formation (Figure S4) while no absorption modification were observed in absence of Aβ (Figure S3) indicating that the $Cu^{II}(AH)_{3N}$ complex was formed during the dead-time of the stopped-flow spectrophotometer. An (almost) isosbestic point is seen at λ = 580 nm, indicating that mainly two species are predominant in solution corresponding to

Cu^{II}(Aβ) and $Cu^{II}(AH)_{3N}$. Hence, no (few) $Cu^{II}(AH)_{2N}$ intermediate has the time to accumulate in solution in line with the formation of the $Cu^{II}(AH)_{2N}$ being the rate the limiting step.

For AAH the presence of Aβ leads to a decrease in the rate of the overall reaction as well and the formation of the $Cu^{II}(AAH)_{4N}$ complex becomes extremely slow. In the studied time window (6s) only about 20-30% of $Cu^{II}(AAH)_{4N}$ complex is formed with 1 equiv. of Aβ. An isosbestic point is seen at λ = 560 nm, indicating that only two species are predominant in solution Cu^{II}(Aβ) and $Cu^{II}(AAH)_{4N}$. Hence, no $Cu^{II}(AAH)_{2N}$ has the time to accumulate in solution in line with the formation of the $Cu^{II}(AAH)_{2N}$ being the rate the limiting step.

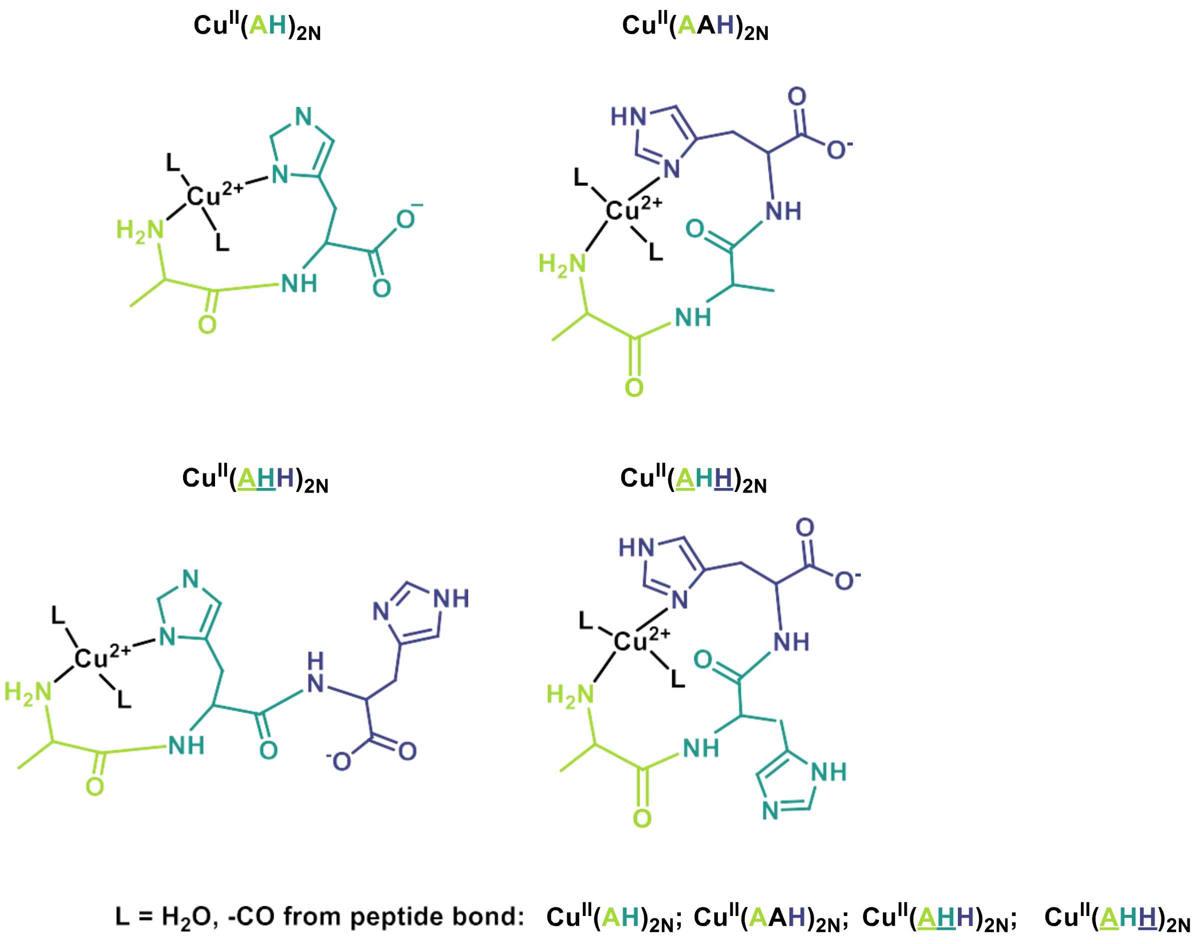

Scheme S1. Proposed coordination sites in the intermediates 2N forms for the AH, AAH and AHH peptides.

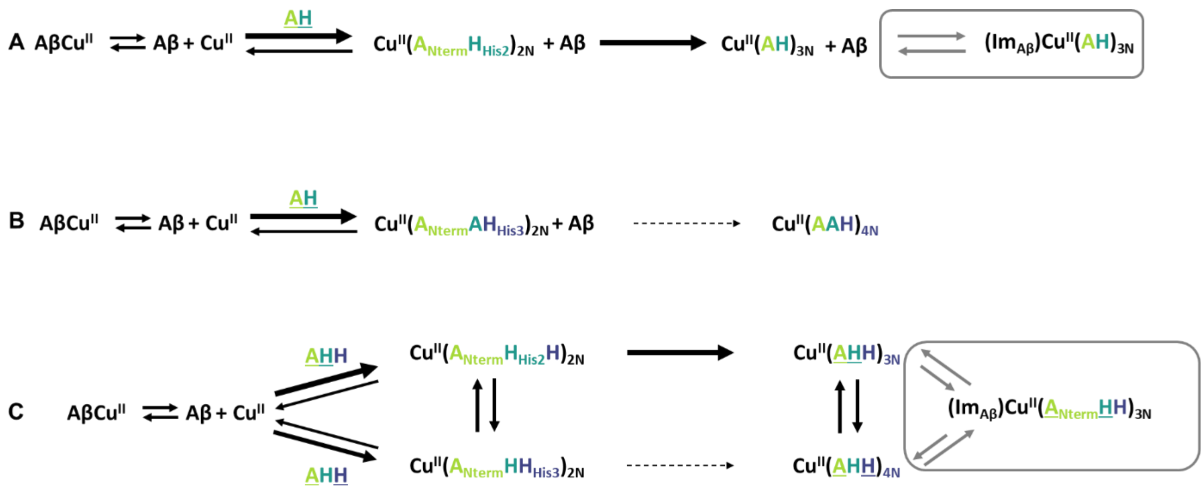

Scheme S2. Proposed mechanism corresponding to Cu^{II} capture out from Aβ by AH (A), AAH (B) and AHH (C). The thickness of the black arrows mirrors the rate of the reaction (thicker the arrow, faster the reaction is & dotted arrows corresponds to the slowest rate). In plain line boxes, are equilibria between Aβ and the Cu^{II}(AH) or $Cu^{II}(AHH)_{3N}$ complexes. These equilibria complete the kinetic scheme 2 in full text (see detailed text below).

Im side-chain from Aβ can form stable ternary species with the $Cu^{II}(AH)_{3N}$ (reaction a) and $Cu^{II}(AHH)_{3N}$ (reaction d), as previously described in case GHK and Aβ[103] or AHH and imidazoles rings.[104] This has no impact on the kinetic of the formation of the stable complexes 3N and 4N complexes. In case of AH, this leads to the formation of a 3N + 1N complex (Scheme S3). Based on literature data, a 50/50 ratio of the ternary species is expected at 500 µM concentration.[62] In case of AHH, this will decrease the proportion of the 4N species formed by less than 10% at 500 µM concentration, based on literature data.[104] At the concentration where ROS are performed the formation of the stable ternary species will be extremely weak (<5%) and thus no impact on ROS is expected due to their formation.

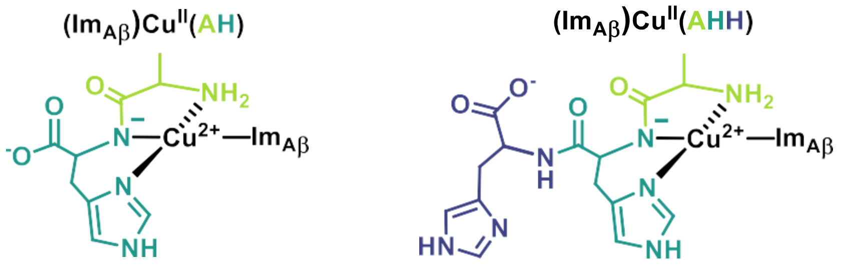

Scheme S3. Proposed coordination sites in the ternary species obtained upon AH and AHH addition to Cu^{II}(Aβ). In case of AHH the ternary species is minor.

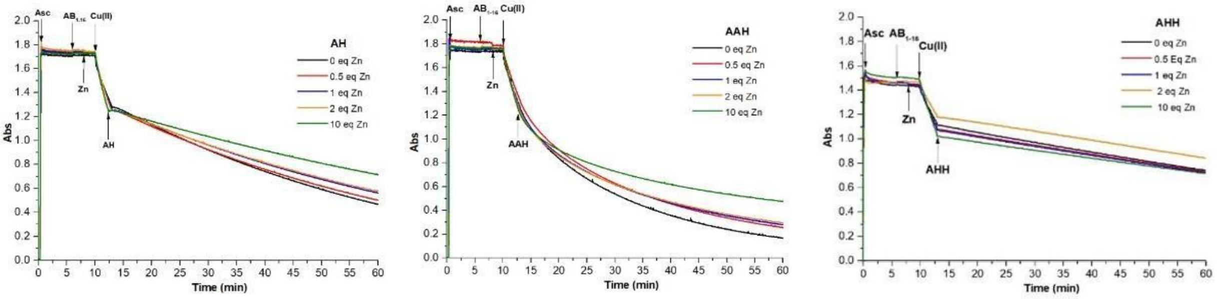

Figure S5. Ascorbate consumption (measured at λ = 265 nm) as a function of time. Ascorbate was added first, followed by Aβ at 6 minutes, Zn^{II} at vario ratios (versus Cu^{II}) at 8 min and Cu^{II} at 10 minutes, and finally the three peptides (AH: left, AAH: middle and AHH: right) when an absorbance of 1.2 was reached. Measurements were performed in 50 mM HEPES buffer (pH 7.5) at 25 °C with continuous stirring. Final concentrations: [Asc] = 100 µM, [Aβ] = [peptide] = 12 µM, [Cu^{II}] = 10 µM, [Zn^{II}] = 5, 10, 20, 100 µM.

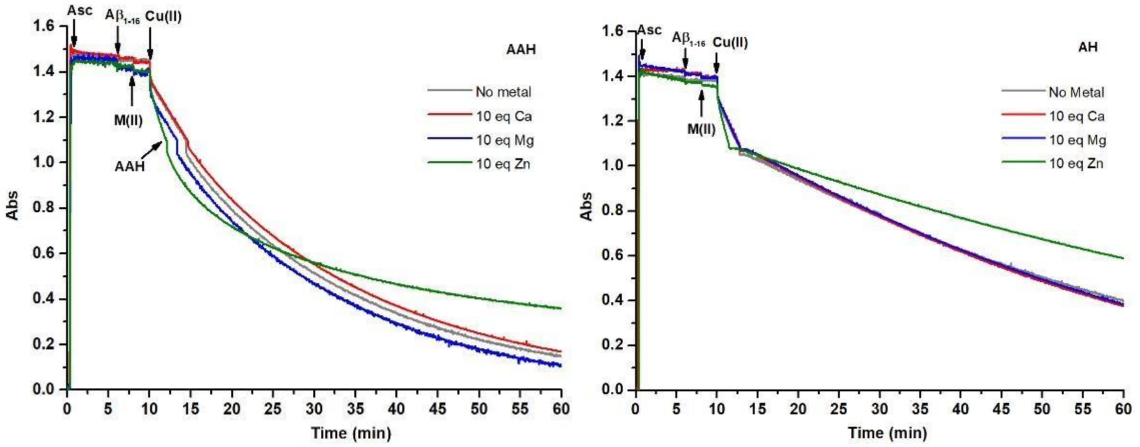

Figure S6. Ascorbate consumption (measured at λ = 265 nm) as a function of time. Ascorbate was added first, followed by Aβ at 6 minutes, Zn^{II} (green), Mg^{II} (blue), Ca^{II} (red) and no ions (grey) at 190 equiv. versus Cu^{II} at 8 min and Cu^{II} at 10 minutes, and finally the AH (left) or the AAH peptide (right) when an absorbance of 1.2 was reached. Measurements were performed in 50 mM HEPES buffer (pH 7.5) at 25 °C with continuous stirring. Final concentrations: [Asc] = 100 µM, [Aβ] = [peptide] = 12 µM, [Cu^{II}] = 10 µM, [Zn^{II}] = [Ca^{II}] = [Mg^{II}] = 100 µM.

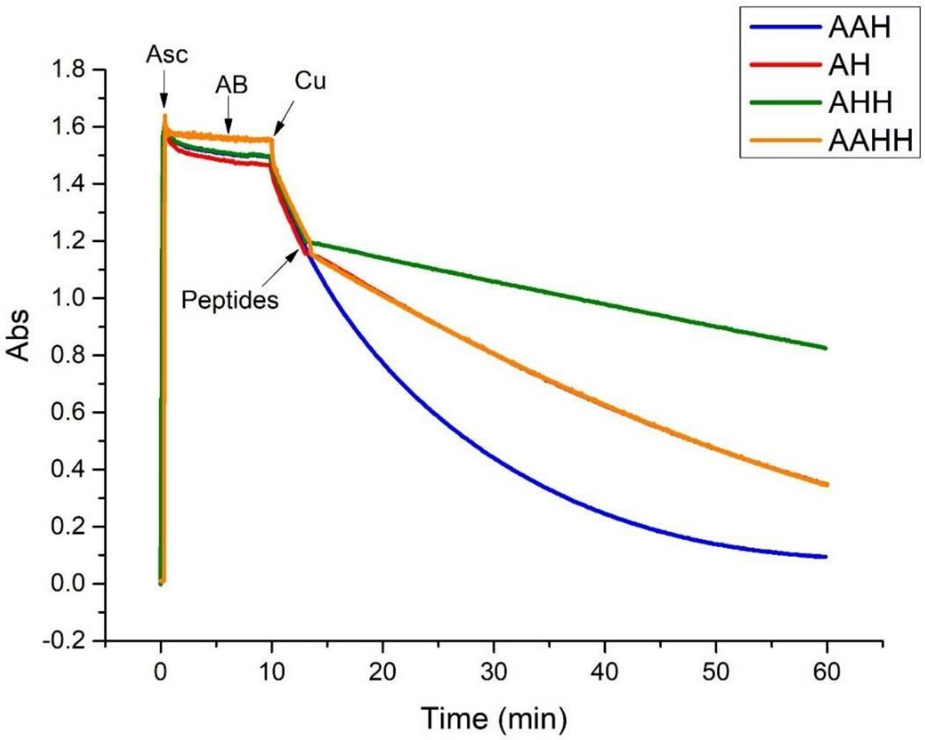

Figure S7. Ascorbate consumption (measured at λ = 265 nm) as a function of time. Ascorbate was added first, followed by Aβ at 6 minutes, Cu^{II} at 10 minutes, and finally the four peptides (AH: red, AAH: blue, AHH: vert and AAHH: orange) when an absorbance of 1.2 was reached. Measurements were performed in 50 mM HEPES buffer (pH 7.5) at 25 °C with continuous stirring. Final concentrations: [Asc] = 100 µM, [Aβ] = [peptide] = 12 µM, [Cu^{II}] = 10 µM. Note that red and orange curves superimpose.

References.

(103) Beuning, C. N.; Zocchi, L. J.; Malikidogo, K. P.; Esmieu, C.; Dorlet, P.; Crans, D. C.; Hureau, C. Measurement of Interpeptidic Cu II Exchange Rate Constants of Cu II-Amyloid-β Complexes to Small Peptide Motifs by Tryptophan Fluorescence Quenching. Inorg. Chem. 2021, 60, 7650-7659.

(104) Gonzalez, P.; Bossak-Ahmad, K.; Vileno, B.; Wezynfeld, N. E.; El Khoury, Y.; Hellwig, P.; Hureau, C.; Bal, W.; Faller, P. Triggering Cu-coordination change in Cu(ii)-Ala-His-His by external ligands. Chem. Commun. 2019, 55, 8110-8113.

(62) Bossak-Ahmad, K.; Wiśniewska, M.D.; Bal, W.; Drew, S.C.; Frączyk, T. Ternary Cu(II) Complex with GHK Peptide and Cis-Urocanic Acid as a Potential Physiologically Functional Copper Chelate. *Int. J. Mol. Sci.* **2020**, *21*, 6190.
